# Detection of Tioman Virus in *Pteropus vampyrus* Near Flores, Indonesia

**DOI:** 10.3390/v13040563

**Published:** 2021-03-26

**Authors:** Susan M. Tsang, Dolyce H. W. Low, Sigit Wiantoro, Ina Smith, Jayanthi Jayakumar, Nancy B. Simmons, Dhanasekaran Vijaykrishna, David J. Lohman, Ian H. Mendenhall

**Affiliations:** 1 Division of Vertebrate Zoology, American Museum of Natural History, New York, NY 10024, USA; stsang@amnh.org (S.M.T.); simmons@amnh.org (N.B.S.); 2 Zoology Division, National Museum of Natural History, Manila 1000, Philippines; dlohman@ccny.cuny.edu; 3 Ph.D. Program in Biology, Graduate Center, City University of New York, New York, NY 10016, USA; 4 Biology Department, City College of New York, City University of New York, New York, NY 10031, USA; 5 Programme in Emerging Infectious Diseases, Duke-National University of Singapore Medical School, Singapore 169857, Singapore; dolyce.low@u.nus.edu (D.H.W.L.); jayanthi.jayakumar@duke-nus.edu.sg (J.J.); 6 Graduate School for Integrative Sciences and Engineering, National University of Singapore, Singapore 119077, Singapore; 7 Museum Zoologicum Bogoriense, Research Center for Biology, Indonesian Institute of Sciences, Cibinong, West Java 16911, Indonesia; wiantoro@gmail.com; 8 Health and Biosecurity, The Commonwealth Scientific and Industrial Research Organization, Black Mountain, ACT 2601, Australia; Ina.Smith@csiro.au; 9 School of Public Health, Li Ka Shing Faculty of Medicine, The University of Hong Kong, Hong Kong, China; veej@hku.hk; 10 SingHealth Duke-NUS Global Health Institute, SingHealth Duke-NUS Academic Medical Centre, Singapore 168753, Singapore

**Keywords:** bat-borne virus, flying foxes, Indonesia, next generation sequencing, paramyxovirus, *Pteropodidae*

## Abstract

Diverse paramyxoviruses have coevolved with their bat hosts, including fruit bats such as flying foxes (Chiroptera: Pteropodidae). Several of these viruses are zoonotic, but the diversity and distribution of *Paramyxoviridae* are poorly understood. We screened pooled feces samples from three *Pteropus vampyrus* colonies and assayed tissues, rectal swabs, and oral swabs from 95 individuals of 23 pteropodid species sampled at 17 sites across the Indonesian archipelago with a conventional paramyxovirus PCR; all tested negative. Samples from 43 individuals were screened with next generation sequencing (NGS), and a single *Pteropus vampyrus* collected near Flores had Tioman virus sequencing reads. Tioman virus is a bat-borne virus in the genus *Pararubulavirus* with prior evidence of spillover to humans. This work expands the known range of Tioman virus, and it is likely that this isolated colony likely has sustained intergenerational transmission over a long period.

## 1. Introduction

Bats (Mammalia: Chiroptera) are unique animals with ecological, immunological, and behavioral attributes that set them apart from other mammalian orders [1]. Bats comprise approximately one-fifth of all extant mammal species and are the only taxon capable of powered flight [2]. Many species roost in large colonies, some of which number more than one million individuals [3], and many bats are unusually long-lived in relation to their body size [4]. Bats are natural reservoirs for a diverse pool of viruses, including several that are zoonotic and able to infect humans [5]. These emerging zoonotic viruses typically originate from areas with relatively high mammal diversity, high human population density, and anthropogenic disturbance [6,7]. Deforestation, forest fragmentation, and agricultural intensification can alter wild habitats, leading to increased opportunities for virus spillover from bats to humans [8,9]. 

Bats host numerous genera in the virus family *Paramyxoviridae*, and proactive discovery of novel paramyxoviruses and their natural reservoir host species benefits public health [10]. Paramyxoviruses are single-stranded, negative-sense RNA viruses in the order Mononegavirales. The genomes of paramyxoviruses are between 15,000–20,000 nucleotides in length, and all have nucleocapsid, phosphoprotein, matrix, fusion, attachment, and large polymerase genes [11]. The large polymerase gene (L) is conserved within *Paramyxoviridae* and is therefore a target for diagnostic, family-level PCR primers [12]. This diverse virus family infects mammals, birds, reptiles and fish, and its four subfamilies include medically important species [13]. The subfamily *Avulavirinae* infects birds and includes the Newcastle disease virus that infects domestic poultry, while the subfamily *Metaparamyxovirinae* consists of a single species that infects fish. The subfamily *Orthoparamyxovirinae* includes several viral genera, including *Morbillivirus* (canine distemper virus and measles virus), *Respirovirus* (human parainfluenza viruses 1 and 3), and *Henipavirus* (Hendra virus and Nipah virus) [11]. The remaining subfamily *Rubulavirinae* includes the human parainfluenza viruses 2 and 4, mumps virus, and several recently discovered bat-borne paramyxovirus such as Achimota virus, Hervey pararubulavirus, Teviot virus, Menangle virus, Sosuga virus, and Tioman virus [14,15].

Flying foxes in the genera *Pteropus* and *Acerodon* include the world’s largest bats (forearm size >89 mm and body mass up to 1600 g) and with 70 species, they constitute over a third of all species in the family Pteropodidae [16,17]. Indonesia is home to about a third of all flying fox species (21 *Pteropus* spp. and 3 *Acerodon* spp.), yet only a handful of studies have explored in detail their coevolutionary and ecological histories with viruses [10]. Most flying foxes are declining throughout Indonesia due to hunting, land conversion resulting in loss of roosting or foraging sites, or the combination of both [18]. As a result of this encroachment, many flying foxes have often been pushed to live at the edges of human settlements or in small refugia in a mosaic of developed landscapes [19,20], leading to an increased probability of transmission; however, no bat-borne zoonotic spillovers having been identified in Indonesia to date.

Pteropodid bats—particularly flying foxes—have received significant attention from virologists because they are natural reservoirs of Hendra virus and Nipah virus [21]. Such surveys are especially critical in understudied tropical regions with potentially high levels of human-bat contact, such as insular Southeast Asia. To better understand viral diversity in flying foxes across the Indonesian archipelago, we used Illumina sequencing to screen tissue samples collected for a project aimed at understanding the molecular phylogeography of *Pteropus* [22], in addition to testing both fecal samples and tissues with a family-specific paramyxovirus PCR.

## 2. Materials and Methods

**Figure 1:**
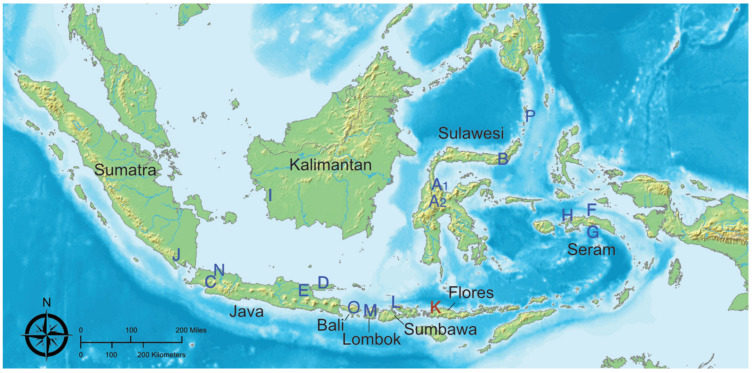
Locations of 17 sites across the Indonesian archipelago where tissues from pteropodid bats were sampled for viral screening. Location K is the site of the *Pteropus vampyrus* positive for Tioman virus.

Tissue samples, rectal swabs, and oral swabs were obtained from wild, adult individuals in Indonesia and were captured from 17 sites throughout Indonesia using either mist nets or traditional hunting methods over the course of 2012–2014 (Figure 1) [19]. We sampled 95 individuals from 23 species, obtaining a total of 262 samples that included multiple sample types from each individual (Table 1).

Samples were collected from nine species of wild-caught flying foxes (*Acerodon celebensis*, *A. mackloti*, *Pteropus alecto*, *P. chrysoproctus*, *P. hypomelanus*, *P. lombocensis*, *P. ocularis*, *P. temminckii*, and *P. vampyrus*) and 14 smaller fruit bats (*Chironax melanocephalus*, *Cynopterus* sp., *Dobsonia* sp., *D. cf. peronii*, *D. moluccensis*, *D. viridis*, *Eonycteris spelaea*, *Macroglossus minimus*, *Nyctimene* sp., *Rousettus* sp., *R. amplexicaudatus*, *R. celebensis*, *R. linduensis*, and *Syconyteris australis*) (Table S1). Members of three of flying fox taxa (*A. celebensis*, *P. alecto*, *P. vampyrus*) were recognized as belonging to multiple subspecies or found across a broad geographic range, and representative samples were taken from each established day roost. Habitats ranged from tracts of pristine mangrove forest to fragments of lightly disturbed secondary forest near human settlement. In addition, eight *P. alecto* fecal samples were collected in Central Sulawesi during 2012, and 91 *P. vampyrus* fecal samples were collected from two sites in West Java during 2012–2013 (Table S2).

Samples were preserved in RNA*later* (ThermoFisher, Waltham, MA, USA) and kept on wet ice until transfer to a −20 °C freezer. Voucher specimens were preserved in the collections at the Museum Zoologicum Bogoriense at the Indonesian Institute of Sciences in Cibinong, Indonesia (Table S1). Bat capture, handling, and sampling methods were approved by the IACUC committee at City College of New York (Protocol No. 896.2 to D. J. Lohman and S. M. Tsang). Permission for research and fieldwork was granted by the Indonesian Ministry of Research and Technology/National Research and Innovation Agency (RISTEK-BRIN, formerly RISTEK) and the Ministry of Environment and Forestry. 

### PCR, Next Generation Sequencing and Sequence Analysis

Nucleic acids were extracted from bat tissues at the Australian Animal Health Laboratory in Geelong, Victoria. Carbide beads and 300–500 uL of AVL Buffer (Qiagen, Hilden, Germany) were added to each sample and these were homogenized in a Mini-Beadbeater (BioSpec, Bartlesville, OK, USA). The samples were centrifuged for 5 min at 13,000 rpm, and RNA was extracted from 180 µL of the supernatant using a MagMax-96 Viral RNA Isolation Kit (ThermoFisher, Waltham, MA, USA) per the manufacturer’s instructions. RNA was eluted into 50 µL buffer and quantitated with a NanoDrop Spectrophotometer (ThermoFisher, Waltham, MA, USA) to confirm successful RNA extraction. RNA was stored at −80 °C until shipment to Duke-NUS Medical School for conventional PCR and Illumina sequencing. cDNA was synthesized from extracted tissue and feces RNA using Superscript III Reverse Transcriptase (Thermo-Fisher, Waltham, MA, USA) and screened for paramyxoviruses with a hemi-nested conventional PCR [12]. A paramyxovirus positive control plasmid was produced by ligating a PIV4B amplified PCR product into the pGEM^®^-T Easy Vector (Promega Corporation, Madison, WI, USA). The selected clone was validated by restriction digestion and PCR amplification, followed by expansion in maxi culture and purification (NucleoBond Maxi Plus EF, Macherey-Nagel, Düren, Germany) to produce a stock control.

Liver, lung, and kidney tissue samples from 43 bats were pooled based on species and location (Table S3). A total of 200 µL of pooled materials from each set were extracted using Direct-zol™ RNA MiniPrep kit with TRI-Reagent (Zymo Research Corporation, Irvine, CA, USA) per manufacturer’s instruction. RNA was subjected to in-column DNase I (New England BioLabs Inc., Ipswich, MA, USA) digestion as instructed in the Direct-zol™ RNA MiniPrep manual. Extracted and DNase I digested RNA were treated with Ribo-Zero™ Gold Epidemiology Kit (San Diego, CA, USA) following the manufacturer’s protocol. Sequencing cDNA libraries of 260 bp were constructed using a TruSeq^®^ RNA Exome kit (Illumina; San Diego, CA, USA) following the manufacturer’s protocol. Library quality was assessed with an Agilent Bioanalyzer High Sensitivity DNA Assay and sequenced on an Illumina HiSeq2000 platform using a 76 bp paired-end run.

FASTQ files were examined with FastQC to determine quality [23]. Trimming was performed with Trimmomatic-0.3.2 to remove adapters, low quality bases (Q = 20 with a sliding window of 4), and reads smaller than 50 bp [24]. Taxonomic read classification was assigned with DIAMOND using a local National Center for Biotechnology Information (NCBI) nr (non-redundant) protein database [25]. DIAMOND outputs were processed to generate taxonomic classification with MEGAN6 using a naïve least common ancestor algorithm with a minimum score of 50, a max expected score of 0.01 and a top percent of 10 [26]. Paramyxovirus reads were exported, de novo assembled and mapped to a Tioman virus reference genome (NC_004074) in Geneious 11.1.2 (Biomatters Ltd., Auckland, New Zealand) [27]. A 729 basepair alignment was generated using MAFFT with representative L gene sequences from several paramyxoviruses downloaded from NCBI GenBank and two 101 bp reads from the NGS data [28]. Maximum likelihood phylogenetic analysis was performed using IQ-TREE [29]. The best-fit nucleotide substitution model was selected with ModelFinder using the Bayesian information criterion, and branch support was estimated using 2000 ultrafast bootstrap replicates [30]. To infer the robustness of the phylogeny, we performed a likelihood mapping analysis using IQ-TREE for the L gene sequence alignment, including members of the *Rubulavirinae* subfamily.

## 3. Results/Discussion

No paramyxovirus amplicons were generated in the fecal and tissue samples tested with the paramyxovirus PCR. Genomic evidence of Tioman virus was detected from a pooled kidney, liver and lung sample from a single *Pteropus vampyrus* individual from a small island northwest of Flores (Figure 1, site K). A total of 70,956,245 reads were generated from the NGS reaction (NCBI BioProject PRJNA693365). The majority of these reads were vertebrate in origin (83.19%), while unassigned reads were the second most common (9.37%). Virus reads comprised 0.01% (*n* = 5048) of all reads (Table 2). 

**Figure 2:**
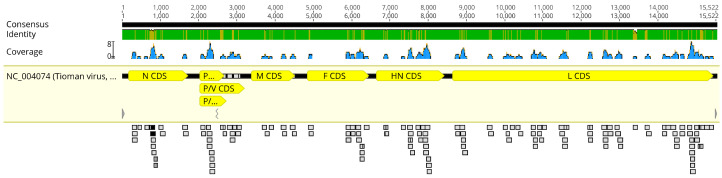
Coverage of next generation sequencing reads from a *Pteropus vampyrus* assembled to a reference genome of Tioman virus (Accession number: NC_004074). Coverage ranged from 0–8 nucleotides across the genome, represented in blue above the annotated reference genome.

**Figure 3:**
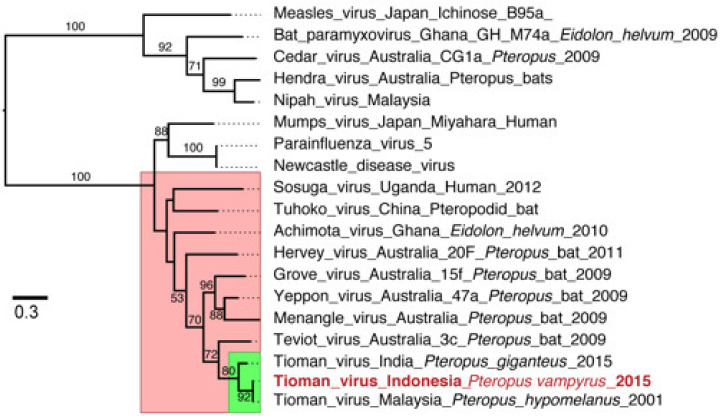
Maximum likelihood phylogeny of the L (polymerase) gene region of paramyxoviruses. The red box denotes sequences from members of the genus *Pararubulavirus* and the green box indicates Tioman virus sequences. Bootstrap values > 50% are indicated at branch nodes. The scale bar represents the nucleotide substitutions per site.

There were 147 paramyxovirus reads, and 143 of these mapped to the Tioman virus reference genome (Figure 2; Table S4). Across the Tioman virus reference genome (15,522 bp; NC_004074), NGS reads covered nearly half the genome (44.3%, 6879 bp) with a nucleotide pairwise identity of 98.5% and a depth of 1–8 nucleotides. There were reads for each gene, including the nucleocapsid gene (41%), phosphoprotein gene (55.1%), matrix gene (36.8%), fusion gene (35.1%), hemagglutinin and neuraminidase gene (39.2%), and the polymerase (L) gene (50.2%). The sequence reads from the *P. vampyrus* formed a monophyletic clade with Tioman virus L gene sequences previously collected from Malaysia and India (bootstrap value = 80). However, the Indonesian sequence was most closely related to viral sequences from a *P. hypomelanus* specimen in Malaysia. Additionally, the L gene sequence of Tioman viruses clustered most closely with other *Pararubulavirus* sequences collected from *Pteropus* species across multiple continents (Figure 3). The likelihood mapping shows that the dataset contains a reasonable amount of tree-likeness as the edges comprise >75% of the values. A bootstrap analysis supported these conclusions with reasonably high support for most nodes (Figure S1).

Tioman virus was first detected in Malaysia on Tioman Island in 2001 during surveillance efforts to detect Nipah virus [15]. This virus was isolated on Vero cells from urine samples from *Pteropus hypomelanus* resulting in cytopathic effect including syncytia. It was confirmed to be a new virus through sequencing and phylogenetic analysis. It is unknown if Tioman virus is pathogenic in humans; however, there is evidence of seroreactivity in humans on Tioman Island (1.8% tested seropositive) [31]. Experimental infections in pigs caused fever and generated neutralizing antibodies, suggesting that they could act as intermediate reservoirs [32]. This genus includes *Menangle pararubulavirus* and *Sosuga pararubulavirus*, which are both pathogenic to humans [33].

Evidence of Tioman virus infection in bats has now been detected in six countries. Species in the genus *Pteropus*, the natural reservoir, range from Oceania, through Australia and tropical/subtropical Asia, to Madagascar [22]. A study in Madagascar detected antibodies against Tioman virus in two out of 349 *Pteropus rufus* tested (0.57%) and in one additional pteropodid bat (*Rousettus madagascariensis*) [34]. Tioman virus was isolated from kidney tissue of a single *P. giganteus* individual (1.5% of bats screened) captured in Assam, India [35]. In New Guinea, sera from 66 *P. conspicillatus* bats were tested for Tioman virus-specific antibodies, and 38% were seropositive [36]. A recent survey in Adelaide, Australia, detected serological evidence of Tioman virus in *P. poliocephalus* [37]. Henipavirus outbreaks and exposure in humans or other animal hosts are concordant with the distribution of their bat reservoirs; however, there is no evidence of previous bat-borne paramyxovirus outbreaks in Indonesia [38]. Our detection of this virus in the Indonesian Archipelago affirms that this virus is likely endemic in *Pteropus* across the genus’ distribution, though questions remain about its endemicity to Australia and the islands of the Pacific.

The remote island northeast of Flores on which the Tioman virus-infected individual was found is a new locality record for *Pteropus vampyrus,* thus expanding the bat’s known range [19]. The phylogenetic distinctiveness of this population suggests lack of recent gene flow with populations on Flores or other islands of the Lesser Sundas [19]. Presence of the virus in such an isolated population suggests that it was likely endemic in the *P. vampyrus* that founded the island’s population. The day roost is within a conservation area on an offshore island with a dense mangrove swamp forest and few beaches. The rugged terrain and the presence of multiple, large (~3 m) Komodo dragons (*Varanus komodoensis*) likely act as a deterrent to hunting or long-term human settlement. These factors may have helped promote population growth and stability, with over 5000 individuals located at a single site. This situation stands in stark contrast to western Indonesian roosts that contend with human settlements and occupy any suitable tree in disturbed landscapes, resulting in spatial fragmentation of large colonies [19].

The relative isolation and apparent stability of the Flores colony combined with its large population size are both likely key contributing factors that promote sustained bat-to-bat transmission of Tioman virus. Isolation on islands often results in ecological feedback that leads to decreased influence from interspecific interactions, but increased influence from intraspecific interactions [39]. Tioman virus might be maintained in this isolated population because bat immunological responses to infection can aid viral persistence, while the annual addition of immunologically naïve pups would allow for sustained transmission within the colony without the need for external introduction from immigrating individuals [40,41]. The paramyxovirus family-specific primers did not detect the virus, indicating that there may have been low amounts of viral DNA in extracts of bat tissues. Increasing viral surveillance may lead to additional viral discovery; there are likely several undiscovered paramyxovirus lineages circulating in bat reservoir hosts across unsampled colonies in Indonesia. Poor knowledge of bat foraging ranges and migration coupled with the difficulty of reaching remote colonies stressed by hunting pressures makes routine surveillance challenging.

## Figures and Tables

**Table 1:** Bat species, province, site code (with reference letter on map), number of bats, and sample types tested for paramyxoviruses with a conventional RT-PCR.

Species	Sampling Location	Site Code	No. bats	Kidney	Liver	Lung	Oral Swab	Rectal Swab	Small Intestine
*Acerodon celebensis*	Central Sulawesi	A1	6	-	6	-	-	3	-
*Acerodon celebensis*	Gorontalo	B	6	-	6	2	4	2	-
*Acerodon mackloti*	Lombok	M	2	2	2	2	3	2	2
*Chironax melanocephalus*	Central Sulawesi	A2	1	-	1	-	-	-	-
*Cynopterus* sp.	Central Sulawesi	A2	5	-	4	-	-	2	-
Dobsonia cf. *peronii*	Lombok	M	1	-	-	-	1	1	-
*Dobsonia moluccensis*	Maluku	H	1	-	1	-	-	-	-
*Dobsonia* sp.	Seram, Maluku	G	2	-	1	-	2	2	-
*Dobsonia viridis*	Maluku	H	3	-	3	-	-	2	-
*Eonycteris spelaea*	West Kalimantan	I	2	-	2	-	-	-	-
*Macroglossus minimus*	Central Sulawesi	A2	2	-	2	-	-	1	-
*Macroglossus minimus*	Seram, Maluku	H	1	-	1	-	-	-	-
*Nyctimene* sp.	Seram, Maluku	G,H	6	-	2	-	4	4	-
*Pteropus alecto*	Central Sulawesi	A1	12	-	11	4	2	5	-
*Pteropus alecto*	Gorontalo	B	4	-	4	-	2	1	-
*Pteropus chrysoproctus*	Seram, Maluku	H	4	4	4	4	4	4	4
*Pteropus hypomelanus*	East Java	E	3	2	2	2	-	3	2
*Pteropus hypomelanus*	Sangihe Islands	P	3	-	3	-	3	2	-
*Pteropus lombocensis*	Lombok	M	1	1	1	1	1	1	1
*Pteropus ocularis*	Seram, Maluku	F	1	1	1	1	1	1	1
*Pteropus temminckii*	Seram Maluku	F,G	3	3	3	2	3	3	3
*Pteropus vampyrus*	Bali	O	1	-	-	-	1	1	-
*Pteropus vampyrus*	East Java	E	2	2	2	2	2	2	2
*Pteropus vampyrus*	Flores	K	2	2	2	2	2	2	2
*Pteropus vampyrus*	South Sumatra	J	2	2	2	2	2	2	2
*Pteropus vampyrus*	West Java	C	4	1	4	2	4	4	1
*Pteropus vampyrus*	West Kalimantan	I	2	2	2	2	2	2	2
*Pteropus vampyrus*	Sumbawa	L	1	1	1	1	1	1	1
*Rousettus amplexicaudatus*	Seram, Maluku	H	2	-	1	-	1	1	-
*Rousettus celebensis*	Central Sulawesi	A2	1	-	1	-	-	-	-
*Rousettus linduensis*	Central Sulawesi	A2	1	-	1	-	-	-	-
*Rousettus* sp.	Central Sulawesi	A2	4	-	3	-	-	2	-
*Syconycteris australis*	Seram, Maluku	G,H	4	-	2	-	2	3	-
**Total**			95	23	81	29	47	59	23

**Table 2:** Next generation sequencing reads for the *Pteropus vampyrus* bat from Flores (Site K) with Tioman virus sequences. The predominant taxa in the fastq file are presented with the percentage of total reads.

Taxon	Forward Unpaired Reads	Reverse Unpaired Reads	Forward Paired Reads	Reverse Paired Reads	Total
Eukaryota	4,569,303 (78.7%)	803,357 (82.0%)	27,331,682 (85.3%)	27,643,874 (86.3%)	60,348,216 (85.0%)
Vertebrata	4,436,518 (75.3%)	788,376 (80.5%)	26,699,724 (83.3%)	27,103,904 (84.6%)	59,028,522 (83.2%)
Platyhelminthes	5949 (0.1%)	111 (<0.1%)	28,122 (<0.1%)	5914 (<0.1%)	40,096 (<0.1%)
Bacteria	93,262 (1.6%)	11,778 (1.2%)	516,241 (1.6%)	507,629 (1.6%)	1128,910 (1.6%)
Fungi	76 (<0.1%)	5 (<0.1%)	392 (<0.1%)	355 (<0.1%)	828 (<0.1%)
Viruses	446 (<0.1%)	79 (<0.1%)	2293 (<0.1%)	2230 (<0.1%)	5048 (<0.1%)
Herpesvirales	3 (<0.1%)	0 (0%)	72 (<0.1%)	52 (<0.1%)	127 (<0.1%)
Retroviridae	97 (<0.1%)	28 (<0.1%)	889 (<0.1%)	893 (<0.1%)	1907 (<0.1%)
Poxviridae	2 (<0.1%)	1 (<0.1%)	16 (<0.1%)	16 (<0.1%)	35 (<0.1%)
Paramyxoviridae	5 (<0.1%)	4 (<0.1%)	70 (<0.1%)	68 (<0.1%)	147 (<0.1%)
Not assigned	1,011,911 (17.2%)	127,330 (13.0%)	2,916,552 (9.1%)	2,586,915 (8.1%)	6,645,708 (9.4%)
Total Reads	5,890,265	979,360	32,056,464	32,030,156	70,956,245

## Data Availability

Supporting data can be found at the NCBI BioProject PRJNA693365.

## Supplementary Materials

The following are available online at https://www.mdpi.com/article/10.3390/v13040563/s1. Table S1: Species and sample list for conventional PCR screening. Table S2: Museum voucher numbers of bat samples. Table S3: Species and sample list for next generation sequencing libraries. Table S4: Tioman virus sequences by gene from next generation sequencing data. Figure S1: Likelihood mapping for the *Rubulavirinae* dataset comprising 14 sequences of 729 bp.
