# Supplementary figures and images for: Detection of Tioman Virus in Pteropus vampyrus Near Flores, Indonesia
Source: Viruses. 2021 Mar 26;13(4):563. doi: 10.3390/v13040563 (PMC8067168; doi:10.3390/v13040563)

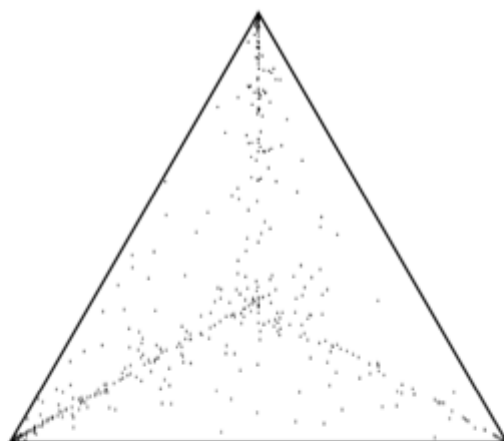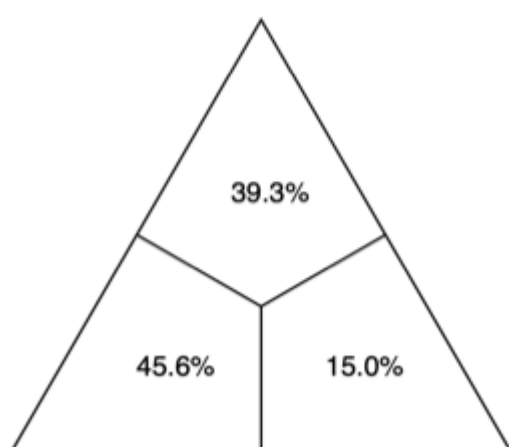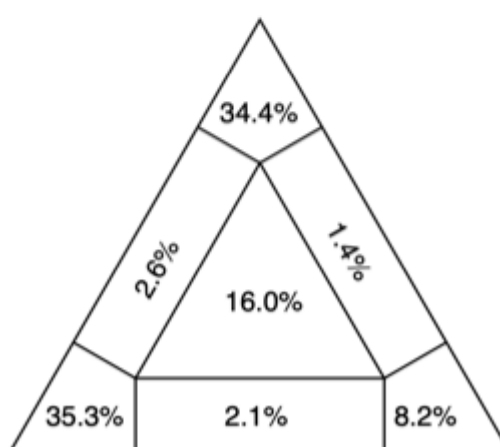

Supplement: Supplementary file 1 [file viruses-13-00563-s001.zip › Figure S1.pdf]
